# Reagent to Detect the Quantity of Urea in the Urine

**Published:** 1853-03

**Authors:** 


*Reagent to detect the quantity of Urea in the Urine.*—Mr. Liebig has recently made known the following prompt and easy way of detecting the quantity of urea in the urine. First prepare a neutral solution of nitrate of mercury in distilled water, to be kept by itself, and add a little of this gradually to the urine to be examined, until no further precipitate takes place. The quantity of the solution necessary to this result is, to a certain point, the measure of the proportion of urea. But the precipitate, which is formed, is composed of one equivalent of nitric acid and three equivalents of bi-oxide of mercury; hence for every equivalent of urea precipitated, there will be three equivalents of free nitric acid in the liquid. But the presence of this free acid interferes with the further formation of the nitrate, so that when precipitation has ceased, there is still more or less urea which has not entered into the new combination, and which can only be precipitated after the free acid has been saturated. This is accomplished by a solution of barytes, which must be gradually added so as not to exceed the limit of saturation. A fresh quantity of the solution of nitrate of mercury must then be added to produce a new precipitate; and thus, by the successive additions of this solution and of the solution of barytes, the whole of the urea contained in the urine will be precipitated. The quantity of the solution of nitrate of mercury used thus forms a sufficiently accurate measure of the quantity of urea in the urine.—*Bull. de Thér.*—*From Gaz. des Hop.*, 37 *Juillet*, 1852.

